# Direct Quantification of Cd^2+^ in the Presence of Cu^2+^ by a Combination of Anodic Stripping Voltammetry Using a Bi-Film-Modified Glassy Carbon Electrode and an Artificial Neural Network

**DOI:** 10.3390/s17071558

**Published:** 2017-07-03

**Authors:** Guo Zhao, Hui Wang, Gang Liu

**Affiliations:** 1 Key Lab of Modern Precision Agriculture System Integration Research, Ministry of Education of China, China Agricultural University, Beijing 100083, China; 15264315915@163.com (G.Z.); wanghui_lunwen@163.com (H.W.); 2 Key Lab of Agricultural Information Acquisition Technology, Ministry of Agricultural of China, China Agricultural University, Beijing 100083, China

**Keywords:** bismuth-film electrode, artificial neural network, square-wave anodic stripping voltammetry, Cu^2+^, Cd^2+^, quantitative detection

## Abstract

In this study, a novel method based on a Bi/glassy carbon electrode (Bi/GCE) for quantitatively and directly detecting Cd^2+^ in the presence of Cu^2+^ without further electrode modifications by combining square-wave anodic stripping voltammetry (SWASV) and a back-propagation artificial neural network (BP-ANN) has been proposed. The influence of the Cu^2+^ concentration on the stripping response to Cd^2+^ was studied. In addition, the effect of the ferrocyanide concentration on the SWASV detection of Cd^2+^ in the presence of Cu^2+^ was investigated. A BP-ANN with two inputs and one output was used to establish the nonlinear relationship between the concentration of Cd^2+^ and the stripping peak currents of Cu^2+^ and Cd^2+^. The factors affecting the SWASV detection of Cd^2+^ and the key parameters of the BP-ANN were optimized. Moreover, the direct calibration model (i.e., adding 0.1 mM ferrocyanide before detection), the BP-ANN model and other prediction models were compared to verify the prediction performance of these models in terms of their mean absolute errors (MAEs), root mean square errors (RMSEs) and correlation coefficients. The BP-ANN model exhibited higher prediction accuracy than the direct calibration model and the other prediction models. Finally, the proposed method was used to detect Cd^2+^ in soil samples with satisfactory results.

## 1. Introduction

Recently, effectively detecting Cd^2+^ has become increasingly important because Cd^2+^ content in water and soil poses a serious threat to ecological systems and public health via the food chain due to its non-biodegradability and toxicity [1,2,3].

Anodic stripping voltammetry (ASV), an electrochemical technique, has been widely used for the analysis of heavy metal ions (HMs) at trace levels because of its extraordinary characteristics, such as good selectivity, portability, low cost, fast analysis speed and excellent sensitivity [4,5,6,7,8]. During the analysis of HMs using ASV, the HMs were electrodeposited onto the electrode surface via an electrodeposition process and then stripped off the electrode surface via a stripping process [9]. The concentration of the HMs was proportional to their stripping peak currents, which flow during the stripping process [10]. In addition, the type of HMs can be identified by the potential at which the stripping initiates. However, the presence of Cu^2+^, which is the most pronounced interference ion, obviously influences the stripping currents of Cd^2+^ [11,12,13], leading to inaccurate detection results. This inhibitory action is presumably due to the formation of intermetallic compounds and competition for active sites on the electrode surface [14].

Numerous studies have been devoted to developing new electrode materials and electrode modifications and have attempted to improve the sensitivity and anti-interference performance of electrodes [15,16,17]. Chemically modifying electrodes effectively improves their sensitivity and anti-interference performance, but the presence of Cu^2+^ still observably diminishes the stripping peak currents of Cd^2+^ [18,19,20]. Adding ferrocyanide to the sample extract solutions can decrease the Cu^2+^ interference to some extent [21,22,23] because insoluble, stable copper-ferrocyanide complexes can form with the help of a ligand [24]. However, these complexes suffer several limitations, i.e., both too much and too little ferrocyanide will influence the Cu^2+^-shielding performance of ferricyanide. Furthermore, adding ferrocyanide requires an optimization process, which would decrease the efficiency of on-site Cd^2+^ detection in real samples.

Although the presence of Cu^2+^ will decrease the stripping peak currents of Cd^2+^, ASV can simultaneously measure the stripping signals of Cd^2+^ and Cu^2+^. Thus, the stripping signals of Cd^2+^ and Cu^2+^ could be used to quantitatively reflect the concentration of Cd^2+^, indirectly reflecting the degree to which Cu^2+^ suppresses the stripping peak current of Cd^2+^. Analysing the measured ASV spectrogram revealed a multivariate nonlinear relationship between the stripping signals of Cd^2+^ and Cu^2+^ and the Cd^2+^ concentration over the concentration range studied. Thus, without further processing, linear models cannot accurately estimate the Cd^2+^ concentration over a wide range using the data from ASV measurements. Therefore, to correctly interpret these results, an interesting option is to use a versatile mathematical tool known as an artificial neural network (ANN) [25]. ANNs are algorithms that are very well suited to process, discover, and interpret nonlinear relationships in databases by creating simple and manageable mathematical models [26]. To inexpensively and easily detect Cd^2+^ using square-wave ASV (SWASV), a Bi-film-modified GCE was used in this study because of its wide potential window, low toxicity, simple preparation and ability to form alloys with many HMs [27,28,29,30].

In this paper, the interference of different concentrations of Cu^2+^ with the stripping peak current of Cd^2+^ was studied, and the shielding effect of various concentrations of ferrocyanide on the Cu^2+^ for the SWASV detection of Cd^2+^ was studied. In addition, a back-propagation ANN (BP-ANN) was developed to process, discover, and interpret the nonlinear relationships between the concentration of Cd^2+^ and the stripping signals of Cu^2+^ and Cd^2+^ and thus create a simple and manageable mathematical model for Cd^2+^ detection. Moreover, the prediction performance of the BP-ANN model, the direct calibration model and other prediction models were investigated and compared to verify the feasibility of the proposed method. To the best of our knowledge, very few reports have combined SWASV and BP-ANN to quantitatively and directly determine the concentration of Cd^2+^ in the presence of Cu^2+^. Consequently, the combination of ANNs and ASV has the potential to serve as a method for detecting and quantifying many different types of HMs in different kinds of natural samples.

## 2. Materials and Methods

### 2.1. Reagents and Instrumentation

Stock solutions of Cu^2+^, Bi^3+^ and Cd^2+^ (1000 mg/L) were obtained from the National Standard Reference Materials Center of China (Beijing, China) and diluted as required. Acetate buffer solution (0.1 M) was used as the supporting electrolyte to supply the deposition and stripping conditions for Cd^2+^ and Cu^2+^. All other chemicals were used without further purification and were of analytical grade. We used Millipore-Q water (18.2 MW) obtained from Beijing Science and Technology Development Co., Ltd. (Beijing, China) for all experiments. Additionally, a CHI660D electrochemical workstation (Shanghai CH Instruments, Shanghai, China) was used to perform SWASV. A counter electrode made of platinum wire, an Ag/AgCl reference electrode and a Bi/glassy carbon working electrode (Φ = 3 mm) were used to build a three-electrode system. A magnetic stir bar was placed into a 25 mL cell to stir the solution used for all electrochemical measurements during the deposition and cleaning steps. The scanning electron microscopy (SEM) and energy dispersive spectroscopy (EDS) analysis were carried out on JSM-6701F field emission scanning electron microscope produced by JEOL Ltd. (Tokyo, Japan).

### 2.2. Preparation of Bi-Film-Modified Glassy Carbon Electrode (GCE)

The GCE surface was polished with 0.05-mm alumina powder, then sequentially rinsed with 1:1 HNO_3_–H_2_O, absolute ethanol and water before modifying the electrode with the bismuth film, and finally was dried under a N_2_ atmosphere. Next, acetate buffer solution (20 mL, 0.1 M, pH 5.0) was added into a beaker, and then Bi^3+^ stock solution was added to achieve a solution containing 600 μg/L Bi^3+^. Then, for the deposition step, the pretreated GCE was placed in the beaker at a potential of −1.2 V (versus Ag/AgCl) for 150 s while stirring the solution to obtain a Bi-film-modified GCE.

### 2.3. SWASV Detection of Cd^2+^ in the Presence of Cu^2+^

Under the optimized conditions, SWASV for the detection of Cd^2+^ was performed as follows: 20 mL of acetate buffer solution (0.1 M, pH 5.0) was added into a beaker, and the stock solutions of Bi^3+^, Cd^2+^ and Cu^2+^ were added to achieve a solution containing 600 μg/L Bi^3+^ and different concentrations of Cd^2+^ and Cu^2+^ ranging from 1 to 50 μg/L. Then, the three-electrode system consisting of the platinum wire electrode, Ag/AgCl electrode and Bi/GCE was placed in the beaker to carry out the following deposition and stripping process. During the deposition process, a magnetic stir bar was used to stir the solution in the beaker, and the deposition of HMs was performed at a potential of −1.2 V for 150 s. Then, after the stripping process was conducted at a frequency of 25 Hz, a voltammogram recorded as the potential was changed from −1.2 to +0.2 V without stirring. The frequency, step amplitude and pulse amplitude were 25 Hz, 5 mV and 25 mV, respectively. After the stripping process, an activation process using a constant potential of 0.31 V for 120 s was carried out to remove the residual bismuth film and metals on the surface of GCE.

### 2.4. ANN Modelling

ANNs, which are a type of machine learning algorithm [26,31], were inspired by biological neural systems and have been widely used in the area of modelling as a nonlinear prediction model due to their remarkable characteristics. For example, ANNs are flexible and do not need a rigid mathematical model, and the parameters for the prediction model can be determined via a learning step [32]. In this study, a BP-ANN was selected and used to discover and interpret nonlinear relationships present in databases due to its wide application, as it is one of the most widely used ANN methods [33,34,35]. The BP-ANN was trained with data from the SWASV spectrogram, which corresponded to known concentration of Cd^2+^ and Cu^2+^, under supervision because training and optimizing this ANN also required target data (in this case, known Cd^2+^ and Cu^2+^ concentrations) [36].

In this paper, a BP-ANN with three types of layers (an input layer, a hidden layer and an output layer) was used to build a nonlinear prediction model based on the data from SWASV voltammograms for the detection of Cd^2+^ in the presence of Cu^2+^ using a Bi-film-modified GCE. There are several nodes in the input layer; these nodes were determined by the number of independent variables that were employed in the model input [37]. As the actual calculation core of the BP-ANN, the hidden layer and output layer, which are composed of neurons, play an important role in the building of the BP-ANN. To determine the network topology that provides the best statistical results, the neuron number in the hidden layer (NNHL) should be optimized adequately.

**Figure 1:**
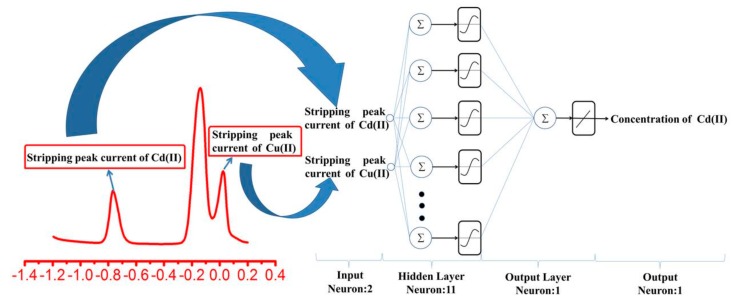
Schematic of the ANN structure used to predict the Cd^2+^ concentration.

The correct definition of the NNHL is very important to the prediction accuracy of BP-ANN because the NNHL at a low level may negatively influence the learning ability of the BP-ANN. However, if the NNHL is too high, the resulting ANN may be over-fitted to the dataset employed [38]. Moreover, the neurons in output layer were determined based on the independent variable that was employed in the model output [39]. In this paper, the three-layer ANN was developed to build a nonlinear model for the prediction of Cd^2+^ concentration in the presence of Cu^2+^. There are two inputs, i.e., the stripping peak current of Cd^2+^ and the stripping peak current of Cu^2+^, and one output, i.e., the concentration of Cd^2+^, in the ANN model, as shown in Figure 1.

Some BP-ANN-related parameters were selected, whereas others were optimized to find the best possible model for estimating the concentration of Cd^2+^ in the presence of Cu^2+^. The best possible model was chosen based on the absolute error (MAE; Equation (1)), root mean square error (RMSE; Equation (2)) and *R*^2^ correlation coefficient (Equation (3)) of the model results. The NNHL, training function, and transfer function were selected as the parameters for optimizing the BP-ANN model. MATLAB R2012b software (MathWorks, Inc., Natick, MA, USA) was used for all ANN-related simulations and calculations.

(1) $$\mathrm{MAE}=\frac{1}{n}\sum_{i=1}^{n}\left|X_{pi}-X_{ai}\right|$$

(2) $$\mathrm{RMSE}=\sqrt{\frac{\sum_{i=1}^{n}{\left(X_{pi}-X_{ai}\right)}^{2}}{n}}$$

(3) $$R^{2}=\frac{n\sum_{i=1}^{n}X_{pi}X_{ai}-\left(\sum_{i=1}^{n}X_{pi}\right)\left(\sum_{i=1}^{n}X_{ai}\right)}{\left(n\left(\sum_{i=1}^{n}{X_{pi}}^{2}\right)-{\left(\sum_{i=1}^{n}X_{pi}\right)}^{2}\right)\left(n\left(\sum_{i=1}^{n}{X_{ai}}^{2}\right)-{\left(\sum_{i=1}^{n}X_{ai}\right)}^{2}\right)}$$

where *n*, *X_pi_*, and *X_ai_* are the total number of predictions and the predicted and actual values (i.e., experimental values), respectively.

### 2.5. Preparation of Soil Samples

The soil samples were obtained from farmland in China. Briefly, the soil samples were dried in an oven for 2 h, then were pulverized on a portable soil crusher and subsequently were sieved through a 200 mm sieve. The soil samples (1 g) were placed in an extraction bottle and extracted with 40 mL of 0.11 M acetic acid. The mixed samples were shaken in an end-over-end shaker for 16 h at room temperature. The mixed samples were subjected to centrifugal sedimentation for phase separation. The heavy metal extracts in the aqueous phase were then filtered with a membrane to remove micro-impurities from the solutions. The exchangeable fractions of heavy metals and carbonate bound heavy metals are more harmful to humans and the environment. The Cd^2+^ that we detected in the soil is in the carbonate bound form. According to the sequential extraction procedure for the speciation of particulate trace metals proposed by A. Tessier et al. [40] in 1979, the soil sample was leached at room temperature with NaOAc solution adjusted to pH 5.0 with acetic acid (HOAc) to obtain the carbonate bound metals. Moreover, according to the results presented in Section 3.1, the maximum stripping peak current of the SWASV-based Bi/GCE appeared at pH 5.0. Considering the abovementioned factors, the pH of the extract solutions was adjusted to 5.0. 

## 3. Results and Discussion

### 3.1. Optimization of Experimental Conditions

**Figure 2:**
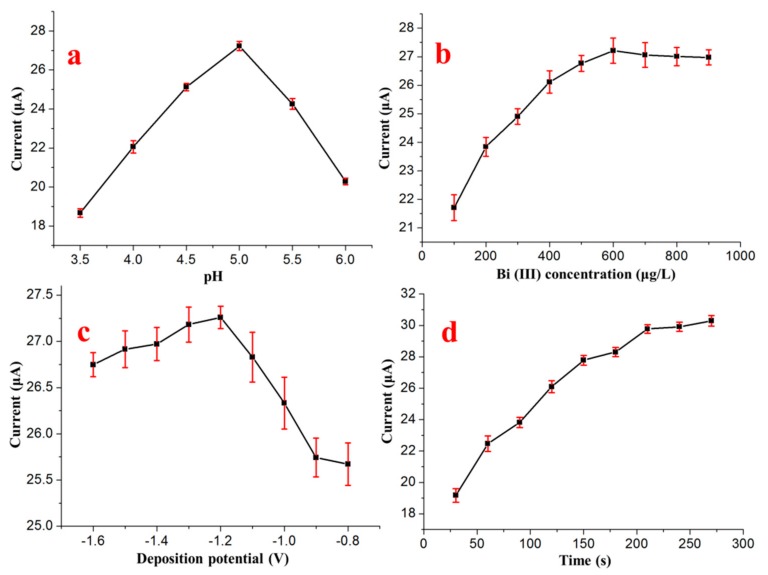
Effects of (**a**) pH, (**b**) Bi^3+^ concentration, (**c**) deposition potential and (**d**) deposition time on the stripping peak currents of 50 μg/L Cd^2+^ in the presence of 20 μg/L Cu^2+^.

To achieve better sensitivity for the determination of Cd^2+^ with the Bi/GCE, different experimental conditions of SWASV were optimized, such as the pH of the supporting electrolyte, concentration of Bi^3+^, deposition potential, and deposition time, as shown in Figure 2. According to the optimization of the experimental conditions, a pH of 5.0, a Bi^3+^ concentration of 600 μg/L, a deposition time of 150 s and a deposition potential of −1.2 V were finally chosen for the following experiments.

### 3.2. Electrochemical Characteristics of the Bi/GCE

**Figure 3:**
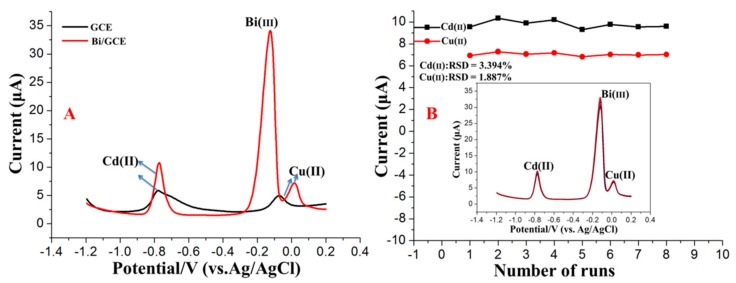
(**A**) SWASV voltammograms of 20 μg/L Cd^2+^ and Cu^2+^ in a 0.1 M acetate buffer solution (pH 5.0) on the GCE and Bi/GCE; (**B**) Stripping current measurements of 20 μg/L Cd^2+^ and Cu^2+^ on the Bi/GCE in a 0.1 M acetate buffer solution (pH 5.0). The insets correspond to data collected from every SWASV response over eight repetitions. RSD: relative standard deviation.

The stripping voltammetry behaviours of Cd^2+^ on the bismuth-film-modified GCE were characterized using a CHI660D electrochemical workstation. The bismuth film was modified in situ with a Bi(III) concentration of 600 μg/L. As probe metal ions, the concentrations of both Cd^2+^ and Pb^2+^ were 20 μg/L. As shown in Figure 3A, the stripping peak signals of Cu^2+^ and Cd^2+^ on the bare GCE were weak and unclear. Comparatively, the Bi/GCE exhibited higher stripping peak signals for Cu^2+^ and Cd^2+^, suggesting that the presence of Bi could promote the reduction of Cu^2+^ and Cd^2+^ because of the unique advantages of the Bi-film-modified electrodes, such as the ability to form alloys with the HMs. Eight repetitive measurements of 20 μg/L Cu^2+^ and Cd^2+^ in acetate buffer solution (pH 5.0, 0.1 M) were performed to verify the reproducibility and stability of the bismuth-film-modified GCE, as shown in Figure 3B. The results indicate that the stripping peak signals of Cu^2+^ and Cd^2+^ on the bismuth-film-modified GCE were reproducible. The relative standard deviations (RSDs) of the eight repetitive measurements were 1.89% and 3.39% for Cu^2+^ and Cd^2+^, respectively. Under the optimum experimental conditions, the bismuth-film-modified GCE exhibited remarkable stability and reproducibility in the stripping analysis of trace levels of Pb^2+^ and Cd^2+^, providing reliable and stable modelling data for training the BP-ANN model.

### 3.3. Influence of Cu^2+^ on the SWASV Detection of Cd^2+^

**Figure 4:**
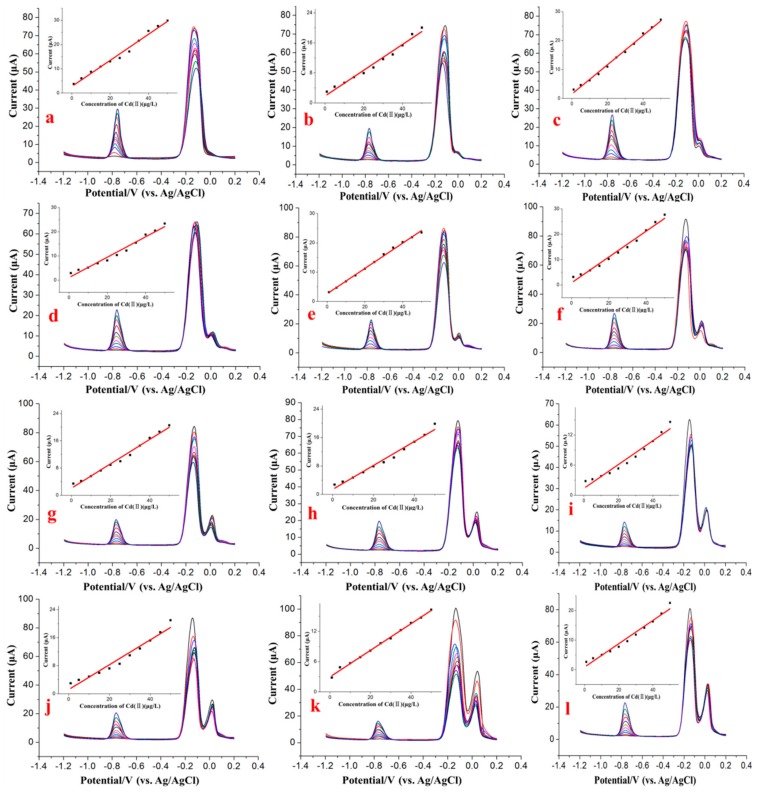
Voltammograms of Cd^2+^ ranging from 1.0 to 50.0 μg/L in the presence of different concentrations of Cu^2+^: (**a**) 0 μg/L, (**b**) 1 μg/L, (**c**) 5 μg/L, (**d**) 10 μg/L, (**e**) 15 μg/L, (**f**) 20 μg/L, (**j**) 25 μg/L, (**h**) 30 μg/L, (**i**) 35 μg/L, (**j**) 40 μg/L, (**k**) 45 μg/L and (**l**) 50 μg/L Cu^2+^. Deposition time: 150 s. Deposition potential: −1.2 V. Concentration of Bi^3+^: 600 μg/L.

Additional SWASV measurements were performed on mixtures of Cu^2+^ and Cd^2+^ to further investigate the influence of various concentrations of Cu^2+^ on the stripping peak current of Cd^2+^. Several binary mixtures of the two species, in which the Cd^2+^ concentration ranged from 1.0 to 50 μg/L and the Cu^2+^ concentration was held constant at a specified value in the range of 0–50 μg/L, were prepared, as shown in Figure 4. Under the optimum conditions, the voltammogram data were obtained from the SWASV detection of the different concentration combinations of Cd^2+^ and Cu^2+^. As shown in Figure 4, the results indicate that there was an approximately linear relation between the concentration of Cd^2+^ and the stripping signals of Cd^2+^ in the range of 1.0 to 50 μg/L, whereas the stripping peak signals of Cd^2+^ were obviously interfered with by the presence of Cu^2+^, even at trace levels.

The Bi film was largely used for modifying the electrode to improve its sensitivity for Cd^2+^ detection, an effect that was attributed to the ability of Bi to “alloy” with Cd^2+^. To the best of our knowledge, there is no obvious relationship between the concentration of Cd^2+^ and stripping currents of Bi^3+^. Cu^2+^ is the most pronounced interference ion, and the presence of Cu^2+^ obviously limits the stripping currents of Cd^2+^. The influence of Cd^2+^ on the stripping currents of Bi^3+^ in the presence of different concentrations of Cu^2+^ is likely due to the formation of intermetallic compounds between Cd, Bi and Cu, which would interfere with the stripping currents of each other.

**Figure 5:**
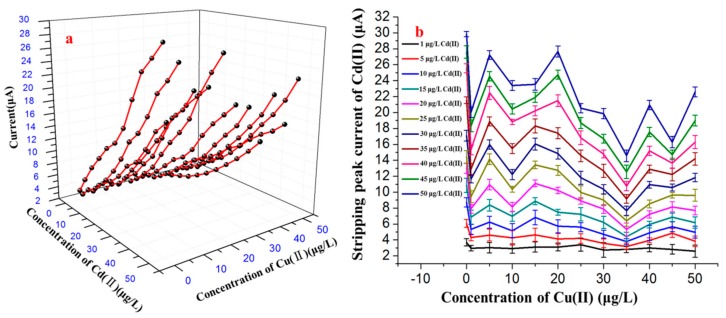
(**a**) Effects of different concentrations of Cu^2+^ on the fitting curve of Cd^2+^. (**b**) Effects of different concentrations of Cu^2+^ on the stripping peak currents of Cd^2+^.

As shown in Figure 5a, the calibration curves obtained with the different concentrations of Cd^2+^ obviously changed as the Cu^2+^ concentration was altered compared with the calibration curve in the absence of Cu^2+^. When the concentration of Cu^2+^ was approximately 35 μg/L, Cu^2+^ most significantly interfered with the stripping response to Cd^2+^. When the concentration of Cu^2+^ was approximately 50 μg/L, Cu^2+^ still interfered with the stripping peak currents of Cd^2+^ but had a more significant effect on the lower concentrations of Cd^2+^ (1.0 to 25 μg/L), as shown in Figure 5b. The standard deviations obtained from five repeated SWASV measurements of the stripping signal are shown as error bars in Figure 5b and were distributed between 0.32 and 0.93. The influence of Cu^2+^ on the stripping current of Cd^2+^ may be due to the generation of intermetallic compounds, which may create competition on the surface of electrode and thereby inhibit the reduction of Cd^2+^ and oxidation of Cd on the surface of electrode during the deposition step and stripping step.

The significance of the linear regression equations and the confidence level were estimated, as shown in Table S1 in the Supplementary Materials. The standard errors of intercept and slope from the linear regression equation can also be seen in Table S1. The results indicated that the linear regression equations were highly significant because the values of “Prob > F” were all less than 0.01. The high significance of the linear regression equations is likely due to the remarkable characteristics of ASV used for the detection of HMs. During the ASV detection of HMs, the HMs were electrodeposited onto the electrode surface with a constant potential; then, the HMs were stripped off the electrode surface electrochemically accompanied by the generation of stripping peak signals. The stripping peak signals that flow during the stripping process showed a good linear relationship with the concentration of the target HMs. In addition, satisfactory experimental errors could be expected because of the high repeatability and stability of the Bi/GCE mentioned above (cf. Section 3.2). However, there were still some problems in real sample detection, although the linear regression equations were highly significant. These problems arose from the presence of various Cu^2+^ concentrations, which will lead to the changes in the intercept and slope of the linear regression equations. More importantly, we did not know the Cu^2+^ concentration in the real samples before the detection of the target HMs; therefore, we did not know which linear equation should be chosen to determine the target HMs in such a situation.

### 3.4. Effects of Ferricyanide on the SWASV Detection of Cd^2+^

**Figure 6:**
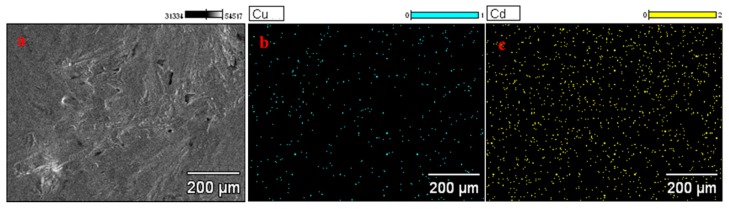
(**a**) SEM image surface morphology of Bi/GCE with the deposition of Cu^2+^ and Cd^2+^. (**b**) and (**c**) Energy dispersive spectroscopy for Cu^2+^ and Cd^2+^ deposited on the surface of GCE.

Cu^2+^ was one of the well-known interference ions that might inhibit the stripping current signals of Cd^2+^, particularly on electroplated bismuth-film electrodes (BiFEs). According to previous reports, this effect may be due to the formation of mixed intermetallic compounds and the undesired deposition of the target metals on electroplated Cu instead of on Bi [41,42]. The formation of intermetallic compounds can seriously interfere with the determination of Cd^2+^ by ASV on BiFEs. The structure characterization of Bi/GCE with the deposition of Cu^2+^ and Cd^2+^ also were evaluated using SEM image, as shown in Figure 6a, which revealed a slightly wrinkled texture. EDS mapping acquired across representative areas of the corresponding Bi/GCE with the deposition of Cu^2+^ and Cd^2+^ reveals that Cu^2+^ and Cd^2+^ has been both electrodeposited onto the electrode surface, as shown in Figure 6b,c, which likely in the form of intermetallic compounds.

**Figure 7:**
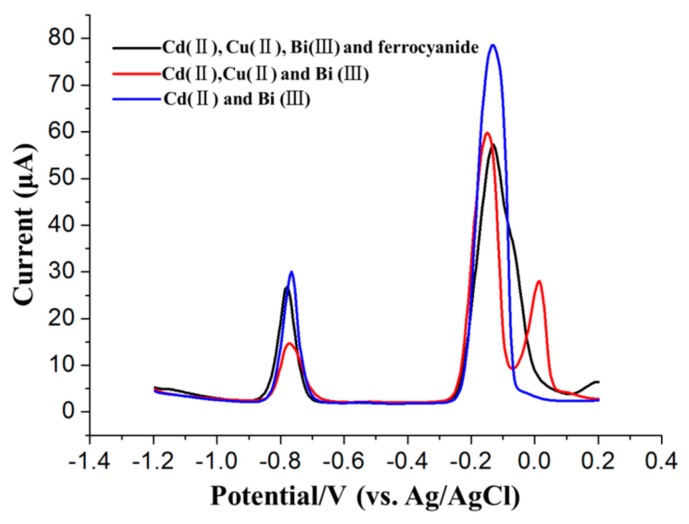
Improvement in the SWASV detection of 45 μg/L Cd^2+^ in the presence of 45 μg/L Cu^2+^ by adding 0.1 mM ferrocyanide.

The interference of Cu^2+^ on the stripping response of Cd^2+^ is commonly alleviated by adding ferrocyanide ions, which form a stable complex with Cu^2+^, as suggested previously [24]. Thus, in this section, 0.1 mM ferrocyanide was used to alleviate the interference of 45 μg/L Cu^2+^ with the stripping signals of 45 μg/L Cd^2+^, thus demonstrating the evident shielding effect of ferricyanide on Cu^2+^, as shown in Figure 7.

**Figure 8:**
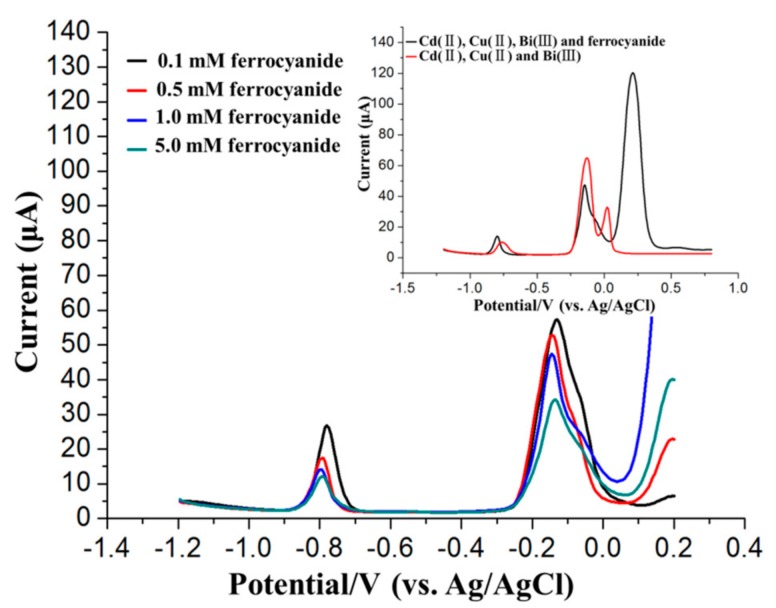
Influence of different concentrations of ferrocyanide on the SWASV detection of 45 μg/L Cd^2+^ in the presence of 45 μg/L Cu^2+^. Inset: SWASV voltammograms of 45 μg/L Cd^2+^ in the presence of 45 μg/L Cu^2+^ before and after adding 1.0 mM ferrocyanide.

In addition, the influence of the ferrocyanide concentration on the SWASV detection of 45 μg/L Cd^2+^ in the presence of 45 μg/L Cu^2+^ was also investigated in this paper. As the ferricyanide concentration increased, the stripping peak currents of Cd^2+^ decreased gradually, as shown in Figure 8, thus demonstrating the obvious influence of the ferricyanide concentration on the Cu^2+^ shielding performance of ferricyanide. 

However, ferrocyanide is not effective unless its concentration is optimized based on specific real samples prior to adding ferrocyanide because copper is commonly found in environmental samples and was observed to be a major interferent in the ASV detection of Cd^2+^ [43,44]. However, the additional optimization process of ferricyanide concentration would doubtlessly decrease the on-site heavy metal detection efficiency. As shown in the upper inset of Figure 7, there are two bumps after the stripping peaks of Bi^3+^ and Cu^2+^, which are due to the presence of ferrocyanide positively shifting the stripping peaks of Cu^2+^ (~250 mV). However, the shift in the stripping peak of Cu^2+^ will not interfere with identifying the stripping peaks of Cd^2+^.

### 3.5. Proposed ANN Model for the Cd^2+^ Detection in the Presence of Cu^2+^

Because of the drawbacks of adding ferrocyanide to mask the suppression effect of Cu^2+^ on the SWASV detection of Cd^2+^, in this work, the SWASV voltammograms measured at various concentrations of Cd^2+^ and Cu^2+^ were used to design a mathematical model that relies on ANNs to assess the concentration of Cd^2+^ in the presence of Cu^2+^.

#### 3.5.1. ANN Model Optimization

A BP-ANN model comprising three layers with two inputs and one output (cf. Figure 1) was developed to predict the concentration of Cd^2+^. Each layer is interconnected by processing elements known as neurons. The model complexity of an ANN is determined by the number of hidden neurons [45]. To ensure the precision of the prediction of the BP-ANN model, the BP-ANN must be trained, which entails using a training dataset to train the network [28]. Furthermore, to verify the prediction accuracy of the proposed BP-ANN model, a testing dataset was used in this study. The sample data of both the training dataset and testing dataset were normalized to improve the performance of the network convergence and eliminate the effect of the magnitude. The input and output variables were normalized based on Equation (4):

(4) $$X_{k}^{\prime }=\frac{\left(x_{max}^{\prime }-x_{min}^{\prime }\right)\left(x_{k}-x_{min}\right)}{\left(x_{max}-x_{min}\right)}+x_{min}^{\prime }$$

where *x_max_* and *x_min_* represent the maximum and minimum values, respectively, and *x'_max_* and *x'_min_* were set to 1 and −1, respectively. The variables were normalized in the range of −1 to 1.

The training parameters such as the training function, transfer function and NNHL play a key role in the simulation efficiency of the network in the process of training [32,46,47]. Thus, determining the optimal combination of these parameters is very important. In this study, the best prediction scheme for the ANN was determined by simulating different training functions, different transfer functions, and different numbers of neurons. As shown in Equation (5), an empirical formula was used to determine the NNHL [48]. In this equation, *n_o_* is the number of output layer neurons, *n_h_* is the NNHL, *n_i_* is the number of input layer neurons, and *l* is a constant that varies from 1 to 10.

(5) $$n_{h}=\sqrt{n_{i}+n_{o}}+l$$

**Figure 9:**
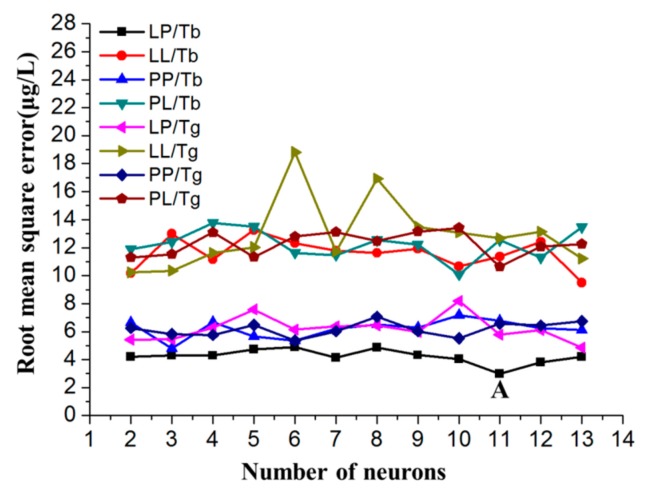
Selection and optimization of the ANN parameters. The transfer functions and training functions were Logsig and Purelin (LP), Logsig and Logsig (LL), Purelin and Purelin (PP), Purelin and Logsig (PL), Trainbr (Tb) and Traingdx (Tg).

According to Equation (5), the NNHL was selected in the range of 2 to 13. Different training functions, such as Trainbr and Traingdx, and transfer functions, such as Purelin and Logsig, were tested to determine the best modelling network. For example, Figure 9 shows that the minimum RMSE corresponds to position “A”, which corresponds to the learning functions Purelin and Logsig for the output layer and hidden layer, respectively, and indicates that these function are optimal for the prediction of Cd^2+^ detection in the presence of Cu^2+^. The trace detection of heavy metals requires the detection of micrograms per litre. Therefore, the ANN model was optimized to achieve as high of a prediction precision as possible, and the modelling network with 11 neurons was used for this study.

#### 3.5.2. Establishment and Validation of the Improved ANN Model

The optimized parameter combination was used to build the ANN model. In total, 81 sets of experimental data were used as the training dataset for training the ANN model, and 40 sets of experimental data were used as the testing dataset to verify the ANN model. The prediction results of the training dataset and testing dataset using the well-trained model are shown in Table S2 and Table S3 (Supplementary Materials). To evaluate the prediction precision of the proposed BP-ANN model, several statistical parameters were used, including the correlation coefficient, RMSE and MAE, as shown in Equations (1)–(3). Table 1 shows the comparison of the prediction performance of the testing dataset and training dataset based on the proposed method, which was conducted by statistical analysis. The reasonable values of *R*^2^, RMSE and MAE for both the testing dataset and training dataset demonstrated that the proposed BP-ANN model was capable of predicting the concentration of Cd^2+^ in the presence of Cu^2+^.

**Figure 10:**
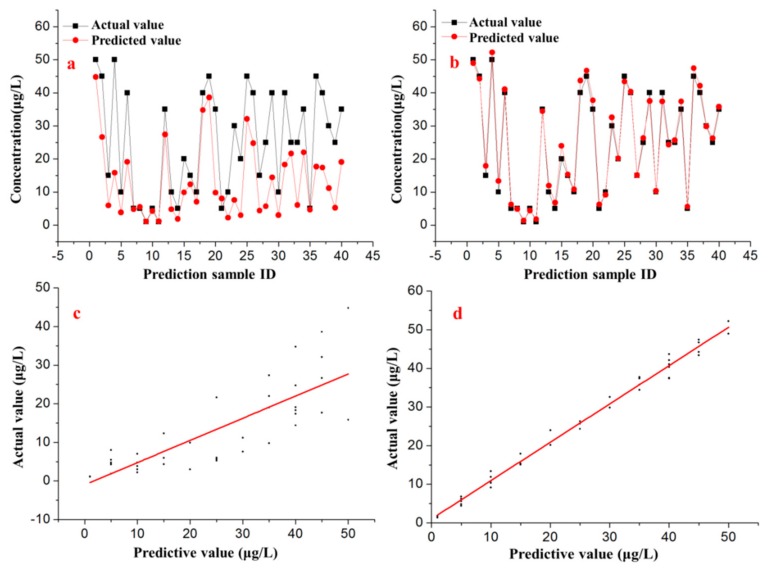
Comparison between the prediction results of the (**a**) direct calibration and (**b**) improved BP-ANN models. Linear regression analysis of the prediction results of the (**c**) direct calibration and (**d**) improved BP-ANN models.

The predicted outputs (concentration of Cd^2+^) of the testing dataset from both the well-trained BP-ANN model and the direct calibration model were compared with the actual values, as shown in Figure 10a,b. The linear regression analysis shown in Figure 10c,d indicated that the predicted values from the BP-ANN model correlated (*R*^2^ = 0.99) more strongly with the actual values than those of the direct calibration model (*R*^2^ = 0.64).

Moreover, to further evaluate the prediction precision and applicability of the proposed method, the statistical parameters, such as *R*^2^, RMSE and MAE, of the direct calibration model (i.e., adding 0.1 mM ferrocyanide before detection), the BP-ANN model and other prediction models were compared, and the results can be seen in Table 2.

Under the optimum conditions, the MAE, RMSE and *R*^2^ of the BP-ANN model were estimated to be 1.42 μg/L, 1.76 μg/L and 0.99, respectively. The corresponding statistical parameters of the direct calibration model were 11.67 μg/L, 14.77 μg/L and 0.64 μg/L, respectively. Further statistical analysis shows that the BP-ANN model exhibited higher prediction accuracy than the direct calibration model and the other prediction models (i.e., binary linear regression and binary nonlinear regression). According to [49], the BP-ANN model having the highest prediction performance among the models may be due to the tendency of ANNs to approximate the nonlinearity of the system.

The effect of other metal cation commonly found in soil samples that could interfere with the SWASV peak currents of Cd^2^^+^ was assessed by comparing the signal currents of a solution of only 10 μg/L Cd^2^^+^ with that of the same solution plus a foreign ion at 10 μg/L. Based on a relative error of greater than 5% being set as the criterion for interference, no interference from the presence of Na^+^, As^3+^, Cr^2+^, K^+^, Ca^2+^, Pb^2^^+^ and Zn^2+^ cations was detected. However, we found that high concentrations of both Pb^2^^+^ and Zn^2+^ could have a significant inhibitory effect on the stripping peak current of Cd^2^^+^.

To further verify its applicability, the proposed method was used to determine the concentration of Cd^2+^ in real soil samples, and the results were compared with those obtained using the standard addition method (SAM), as shown in Table 3. The results show satisfactory recovery results with an average recovery of 98.31%, which indicates that the proposed method is suitable for detecting Cd^2+^ in real samples. Moreover, compared with previous work, the analytical performance of the method developed in this work is comparable or even better [21,22,23,50], as it requires less detection time and has a lower detection cost.

## 4. Conclusions

To overcome the negative suppression effect of Cu^2+^ on the ASV detection of Cd^2+^, a novel method for quantitatively and directly determining the concentration of Cd^2+^ in the presence of Cu^2+^ was proposed in this paper. This method was based on a combination of SWASV and a BP-ANN but did not require further electrode modifications or added ferrocyanide. Importantly, this work not only focuses on the mathematical modelling itself but also integrates potential causative variables (i.e., Cd^2+^ and Cu^2+^) into the models, exploiting the characteristics of ASV, which can simultaneously record the stripping signals of both Cu^2+^ and Cd^2+^. Furthermore, the relationship governing the interference of different concentrations of Cu^2+^ with the stripping voltammetric response to Cd^2+^ was studied. In addition, the interference of different concentrations of ferrocyanide with the SWASV detection of Cd^2+^ in the presence of Cu^2+^ was studied. The statistical results obtained using both the testing dataset and training dataset were reasonable. Comparing the statistical parameters of the direct calibration model, the BP-ANN model and other prediction models shows that the BP-ANN model had the best detection accuracy. Furthermore, for the real samples tested, an average recovery percentage of 98.31% was obtained using the proposed method, which meets the requirements for analysing real samples. The results of this study indicate that combining ASV and machine learning algorithms, such as ANNs, is a promising method to accurately predict the concentration of HMs for food safety supervision, environmental control, and many other applications and fields.

## Figures and Tables

**Table 1:** Prediction results of the BP-ANN model on the training and testing datasets.

Data Set	MAE (μg/L)	RMSE (μg/L)
Training dataset	1.22	1.48
Testing dataset	1.42	1.76

**Table 2:** Comparison of the prediction results of various prediction models.

Prediction Model	MAE (μg/L)	RMSE (μg/L)	*R*^2^
Direct calibration (with ferrocyanide)	11.67	14.77	0.64
Binary linear regression (without ferrocyanide)	4.07	4.92	0.84
Binary nonlinear regression (without ferrocyanide)	2.85	3.77	0.92
ANN (without ferrocyanide)	1.42	1.76	0.99

**Table 3:** Results of the simultaneous detection of Cd^2+^ in soil sample extracts.

Sample No.	Added (μg/L)	Found by SWASV-ANN (μg/L)	Found by SAM (μg/L)	Recovery (%) (SWASV-ANN)	Recovery (%) (SAM)
		Cd^2+^	Cd^2+^	Cd^2+^	Cd^2+^
1	-	4.67	4.96	0.00	0.00
	4.0	8.39	9.14	93.00	101.75
	8.0	12.82	13.06	101.88	98.75
2	-	2.54	2.73	0.00	0.00
	5.0	7.63	7.82	99.60	97.80
	10.0	12.45	12.95	98.10	102.20
3	-	8.16	8.28	0.00	0.00
	10.0	17.98	18.39	98.20	91.10
	15.0	23.02	23.31	99.07	100.20

## Supplementary Materials

The following are available online at http://www.mdpi.com/1424-8220/17/7/1558/s1, Table S1: Calibration equations of Cd^2+^ in the presence of different concentrations of Cu^2+^, Table S2: Experimental design and results of the training dataset, Table S3: Experimental design and results of the testing dataset.
