# Supplementary material for: Direct Quantification of Cd2+ in the Presence of Cu2+ by a Combination of Anodic Stripping Voltammetry Using a Bi-Film-Modified Glassy Carbon Electrode and an Artificial Neural Network
Source: Sensors (Basel). 2017 Jul 3;17(7):1558. doi: 10.3390/s17071558 (PMC5539607; doi:10.3390/s17071558)
Supplement: Supplementary file 1 [file sensors-17-01558-s001.pdf]

Table S1. Calibration equations of Cd^{2+} in the presence of different concentrations of Cu^{2+}.

Concentration of Cu^{2+} (μg/L)	Calibration Linear Equation of Pb^{2+}						
	Slope		Intercept		Adjust R-Square	Prob > F	Confidence Level (%)
	Value	Standard Error	Value	Standard Error			
0	0.54	0.02	2.73	0.59	0.99	6.653×10^{-10}	95
1	0.34	0.02	1.82	0.47	0.98	4.554×10^{-9}	95
5	0.51	0.01	1.53	0.37	0.99	1.803×10^{-11}	95
10	0.42	0.02	1.08	0.65	0.97	1.291×10^{-8}	95
15	0.43	0.01	2.62	0.21	1.00	5.119×10^{-13}	95
20	0.51	0.02	0.82	0.66	0.98	2.952×10^{-9}	95
25	0.36	0.01	2.08	0.38	0.99	5.067×10^{-10}	95
30	0.34	0.02	1.39	0.48	0.98	5.836×10^{-9}	95
35	0.24	0.02	1.36	0.49	0.95	1.609×10^{-7}	95
40	0.35	0.02	1.20	0.67	0.96	7.732×10^{-8}	95
45	0.26	0.01	2.97	0.16	1.00	2.458×10^{-12}	95
50	0.39	0.02	0.90	0.62	0.97	1.567×10^{-8}	95

Table S2. Experimental design and results of the training dataset.

No.	Stripping Peak Current		Concentration (μg/L)		Artificial Neural Network (μg/L)
	Cu^{2+}	Cd^{2+}	Cu^{2+}	Cd^{2+}	Cd^{2+}
1	23.20	3.57	30	5	5.03
2	7.23	5.33	1	10	9.97
3	31.51	10.58	45	30	30.50
4	20.61	17.39	20	35	32.43
5	6.82	9.43	1	25	24.07
6	25.67	2.74	30	1	1.08
7	20.40	10.76	35	40	40.10
8	12.78	13.43	15	25	25.69
9	11.96	16.05	15	30	31.05
10	23.49	3.40	25	1	1.34
11	20.69	14.58	35	50	48.54
12	20.30	9.02	30	25	25.14
13	21.43	2.80	35	1	1.43
14	13.16	10.94	5	20	21.18
15	20.26	7.67	35	30	30.85

16	12.94	6.82	15	10	11.86
17	20.38	12.55	35	45	44.32
18	23.34	7.87	30	20	22.56
19	11.14	21.91	15	45	45.90
20	15.01	20.54	25	50	51.70
21	19.12	16.72	30	45	44.77
22	19.31	14.79	30	40	38.24
23	19.91	19.87	30	50	48.09
24	28.30	14.70	45	45	42.41
25	10.46	12.21	10	30	28.73
26	32.74	18.99	50	45	47.78
27	30.44	2.98	40	1	1.61
28	17.46	9.97	25	25	25.18
29	12.53	11.11	15	20	22.48
30	23.15	4.72	30	10	10.57
31	25.63	20.94	40	50	50.60
32	20.37	10.22	20	20	21.63
33	25.18	8.52	40	25	24.97
34	36.01	8.15	45	20	20.43
35	19.43	12.73	20	25	23.65
36	12.15	15.47	10	35	33.87
37	20.63	9.16	35	35	35.77
38	16.06	4.13	20	5	6.52
39	12.60	2.90	10	1	0.97
40	34.03	2.59	50	1	0.72
41	20.23	21.50	20	40	42.75
42	22.38	12.89	40	35	36.86
43	12.80	15.99	5	30	31.66
44	19.01	14.84	20	30	32.95
45	32.82	6.18	50	15	14.97
46	20.06	7.48	20	15	16.09
47	10.79	10.37	10	25	23.92
48	29.81	16.28	45	50	48.07
49	27.47	10.94	40	30	32.84
50	12.10	8.37	5	15	17.20
51	6.99	2.96	1	1	1.86
52	6.74	12.89	1	35	33.51
53	20.44	3.78	35	10	8.07
54	21.43	5.61	25	10	12.95
55	11.90	5.16	10	10	9.36
56	10.99	8.14	10	20	18.59
57	11.10	23.39	10	50	48.38
58	6.98	15.32	1	40	38.60
59	6.38	20.04	1	50	48.38

60	20.39	4.39	35	15	14.30
61	7.26	11.65	1	30	29.66
62	12.08	6.96	10	15	13.02
63	24.76	5.94	40	15	15.18
64	47.18	4.87	45	5	5.27
65	11.42	20.21	15	40	42.78
66	21.83	6.21	30	15	16.27
67	18.73	14.58	25	35	36.19
68	6.83	7.90	1	20	19.27
69	19.03	24.76	20	45	47.13
70	20.86	10.35	30	30	29.38
71	19.17	27.65	20	50	49.19
72	36.03	12.21	45	35	33.19
73	35.07	3.83	50	5	4.04
74	22.54	4.18	25	5	6.54
75	12.92	8.86	15	15	16.74
76	22.32	3.11	20	1	1.45
77	13.10	27.22	5	50	52.25
78	19.94	5.31	35	20	18.53
79	14.55	3.10	15	1	2.30
80	33.35	7.70	50	20	19.74
81	16.44	18.67	25	45	46.63

Table S3. Experimental design and results of the testing dataset.

No.	Stripping Peak Current		Concentration (μg/L)		Artificial Neural Network (μg/L)
	Cu^{2+}	Cd^{2+}	Cu^{2+}	Cd^{2+}	Cd^{2+}
1	10.75	23.53	15	50	48.98
2	7.12	18.31	1	45	44.25
3	21.68	7.23	25	15	17.93
4	31.06	22.58	50	50	52.21
5	21.65	5.71	20	10	13.37
6	22.13	15.18	40	40	41.06
7	13.07	4.65	15	5	6.22
8	20.69	3.17	35	5	4.84
9	10.23	3.03	5	1	1.39
10	11.08	4.27	10	5	4.41
11	53.47	2.82	45	1	1.78
12	13.36	18.87	5	35	34.44
13	32.65	5.66	45	10	11.93
14	26.15	3.95	40	5	6.79
15	19.21	8.91	25	20	23.96

16	7.16	6.85	1	15	15.33
17	12.30	6.22	5	10	10.82
18	13.93	22.47	5	40	43.71
19	12.67	24.55	5	45	46.71
20	34.43	14.18	50	35	37.75
21	7.24	4.29	1	5	6.28
22	32.73	4.97	50	10	9.11
23	35.21	11.83	50	30	32.57
24	24.04	7.21	40	20	20.17
25	11.14	20.45	10	45	43.43
26	11.50	18.83	10	40	40.34
27	37.64	6.85	45	15	15.09
28	19.99	6.38	35	25	26.31
29	34.40	13.63	45	40	37.54
30	27.09	4.88	40	10	10.33
31	16.73	16.84	25	40	37.38
32	13.93	14.15	5	25	24.29
33	35.61	9.62	45	25	25.67
34	11.58	18.32	15	35	37.39
35	11.77	4.61	5	5	5.59
36	27.05	17.55	40	45	47.43
37	29.81	16.28	45	40	42.14
38	17.81	11.81	25	30	29.87
39	34.03	9.61	50	25	26.31
40	21.63	12.68	30	35	35.79
